# A Two-State Random Walk Model of Sperm Search on Confined Domains

**DOI:** 10.3390/e27050539

**Published:** 2025-05-19

**Authors:** Martin Bier, Maciej Majka, Cameron Schmidt

**Affiliations:** 1 Department of Physics, East Carolina University, Greenville, NC 27858, USA; majkam24@ecu.edu; 2 Department of Biology, East Carolina University, Greenville, NC 27858, USA; schmidtc18@ecu.edu

**Keywords:** sperm cell motion, search trajectories, persistence length

## Abstract

Mammalian fertilization depends on sperm successfully navigating a spatially and chemically complex microenvironment in the female reproductive tract. This process is often conceptualized as a competitive race, but is better understood as a collective random search. Sperm within an ejaculate exhibit a diverse distribution of motility patterns, with some moving in relatively straight lines and others following tightly turning trajectories. Here, we present a two-state random walk model in which sperm switch from high-persistence-length to low-persistence-length motility modes. In reproductive biology, such a switch is often recognized as “hyperactivation”. We study a circularly symmetric setup with sperm emerging at the center and searching a finite-area disk. We explore the implications of switching on search efficiency. The first proposed model describes an adaptive search strategy in which sperm achieve improved spatial coverage without cell-to-cell or environment-to-cell communication. The second model that we study adds a small amount of environment-to-cell communication. The models resemble macroscopic search-and-rescue tactics, but without organization or networked communication. Our findings provide a quantitative framework linking sperm motility patterns to efficient search strategies, offering insights into sperm physiology and the stochastic search dynamics of self-propelled particles.

## 1. Introduction

Mammalian reproduction depends on the ability of sperm cells to locate an egg within the complex microenvironment of the female reproductive tract. Reproductive tracts impose numerous barriers to sperm success, ultimately reducing millions of potential fertilizers to only a few dozen cells that manage to find their way to the ampulla of the oviduct, where fertilization takes place. Although rudimentary forms of navigation, such as chemotaxis and rheotaxis, have been investigated in mammals [1,2], the physiological relevance of these phenomena remains unclear and may be more significant for externally fertilizing organisms that navigate in three dimensions [3,4]. For nearly all eukaryotes, the sperm search for an egg depends, at least in part, on intrinsically controlled motility patterns. Additionally, among species for which forms of taxis have been shown to be important, it is likely that sperm find themselves in regions or conditions where external guidance cues may be weak or absent, highlighting the importance of understanding how intrinsically controlled motility contributes to the search process.

Although the intracellular biochemical mechanisms that control sperm post-ejaculatory behaviors have been well studied in rodents and humans [5], the stochasticity of sperm search owing to complex variation in intrinsically controlled motility patterns remains poorly understood, particularly at the scale of sperm cell populations, which vary from $10^{6}$ to $10^{12}$ cells per ejaculate depending on the species.

Sperm cells are microswimmers that are subject to the physical limitations of life at low Reynolds numbers [6], i.e., inertia plays no role and the motion is overdamped. Furthermore, mammalian sperm move on the epithelial surface of the reproductive tract, and two-dimensional modeling is a reasonable simplification [7]. Sperm motility is driven by active flagellar beating and can be modeled as a random walk [8]. Contemporary computer-aided sperm motility analysis generally focuses on averaged motility patterns on timescales of just a few seconds [9].

A human sperm cell has a width of about 2 μm. Human oviducts are called “fallopian tubes”. Fertilization generally occurs in the lining of a fallopian tube. After release in the vagina, only a fraction of less than one-thousandth of the approximately hundred million sperm cells in a human ejaculate reaches the fallopian tubes. The lining of the fallopian tubes has folds but can still roughly be thought of as the inside of a cylinder. The cylinder is 10 to 14 cm long and about 1 cm in diameter. The egg cell is an ovoid with a diameter of about 0.15 mm. In other words, we are looking at something the size of a pea that is looking for a big beach ball in a search area that is the size of ten football fields.

**Figure 1:**
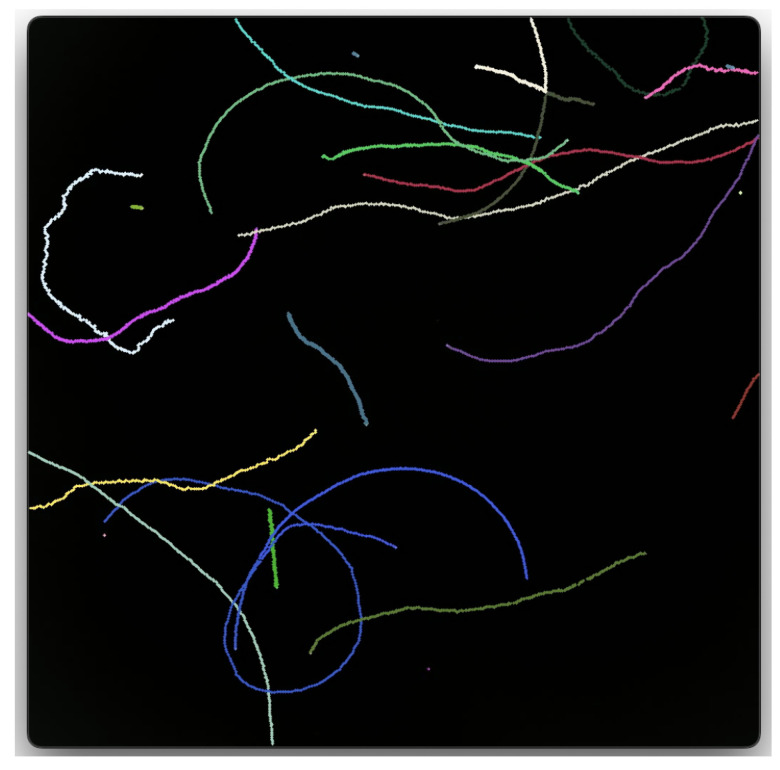
Representative video microscopy trajectories for approximately 20 human sperm over the course of 26 s in a 563 × 563 μm^2^ field of view at 5× magnification at the objective under negative phase contrast. Following 30 min liquefaction at 37 °C in a 5% CO_2_ incubator, sperm were isolated by differential centrifugation in 50% isotonic Percoll. Sperm were resuspended in Biggers, Whitten, and Wiggingham media [15], imaged in a 20 μm deep chambered slide, and temperature was maintained during imaging with a Peltier-heated stage at 37 °C. The sperm cells were observed to move at roughly the same speed, but the persistence lengths differed widely. It is for distinguishability that trajectories have been given different colors.

An important parameter in this study is the persistence length. The persistence length characterizes the sperm’s trajectory, and it can only be observed when the trajectory is followed on a timescale longer than just a few seconds. The persistence length is the distance over which a curve remains directionally correlated, and the concept is also frequently applied in the study of polymers [10,11,12]. We will present the formal definition of the persistence length in Section 2. Suffice it to say at this point that a trajectory with a long persistence length appears as a relatively straight path. A smaller persistence length, on the other hand, corresponds to more frequent changes in direction. Sperm within one ejaculate can exhibit persistence lengths that may differ by orders of magnitude (cf. Figure 1).

Recently, transitions between high-persistence-length movement and low-persistence-length movement have been observed in bovine sperm [13]. This suggests that sperm may transition between mere dispersal and localized search behavior. In confined or semi-confined domains, such as an oviduct in vivo or a culture dish surface in vitro, effective sperm navigation likely requires a strategy that balances rapid traversal across a searchable surface area with a thorough exploration of the local space [8]. The efficiency of sperm search may depend on the transition rates among movement patterns. Transitions between different movement patterns have also been observed in *E. coli* bacteria [14]. In this case, the transitions between the “movement states” were well described with ordinary chemical kinetics (i.e., exponentially distributed dwelling times in states).

Here, we hypothesize that this two-state random walk—switching from a high-persistence length to a low-persistence length—can enhance search efficiency in domains where uniform coverage is essential. We develop a two-state active particle model to explore this hypothesis on a disk with an absorbing boundary, where we let searchers emerge from the center of the disk. In Section 2, we show through numerical simulation how changes in the persistence length can improve the coverage of the search domain. As a baseline, we examine the case of a searcher with a constant persistence length. Next, we study two enhancements. The first one involves a well-tuned transition rate (i.e., exponentially distributed waiting times) to a persistence length that is about an order of magnitude smaller. There is evidence that the change of state can be catalyzed by chemicals that are present in the environment [13,14]. Taking into account this positional guidance, we also study, as a second case, a modified version of our first model: we take the stochasticity out of the switch and let the searching sperm cell change its motility state upon entering the outer section of the disk. In Section 3, we use the diffusion equation to add mathematical rigor to the results from Section 2.

## 2. A Two-State Random Searcher on a Disk

**Figure 2:**
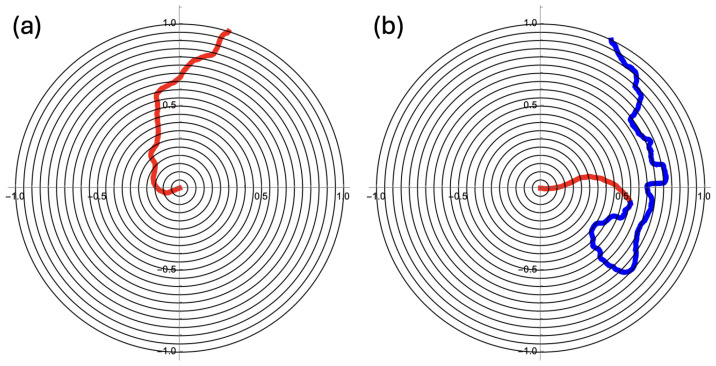
(**a**) The searching particle appears in the center of the unit disk and follows Equation (1) with $v_{0}=1$ and $D_{\theta }=2.0$. The timestep is Δt=0.001, and the edge of the disk is an absorbing boundary. (**b**) Initially, the angular diffusion coefficient is $D_{\theta ,1}=0.67$. There is a transition rate k=2.0 to switch to $D_{\theta ,2}=6.0$. The part of the trajectory before the switch is almost ballistic and is shown in red. The remaining part is shown in blue. With k=2.0 as the transition rate, the switch will, on average, occur close to r=0.5. All numerical work in this article was carried out using the *Mathematica* 13.3 software package.

Our geometric setup is simple. Searching particles appear in the center of a disk and follow a random trajectory until they hit the absorbing boundary at the circumference. We set the disk’s radius at r=1 (cf. Figure 2). Each moving searcher starts out with a large persistence length that is on the order of magnitude of the radius of the disk. The switch is to a persistence length that is about an order of magnitude smaller. We gather statistics for three cases: (*i*) a persistence length that is fixed at half the disk’s radius and does not change, (*ii*) a persistence length that is initially set at one-and-a-half times the disk’s radius, with a transition rate that is set such that the irreversible switch to the smaller persistence length occurs on average at about r=1/2, and (*iii*) persistence lengths as in the second case, but with the irreversible switch occurring at the moment that r=1/2 is first crossed. We consider a search effective if there is no place for the target to “hide” on the disk. In practical terms, this means that all parts of the disk are equally searched. We divide the disk into annuli and numerically investigate. The circular symmetry of this setup keeps the analysis very manageable. In the next section, we set up and analyze a diffusion equation to help us understand the results of the stochastic simulations.

After the sperm is randomly distributed into different parts of the reproductive tract, each sperm performs a local search within a reasonable distance relative to its own body length. Such a search can be well modeled using the disk-with-absorbing-boundary setup described in the previous paragraph.

We model the sperm cell as an active particle that moves at a constant speed $v_{0}$. Fluctuations in the direction of motion θ are accounted for with a diffusion coefficient $D_{\theta }$. This stochasticity is due to active flagellar fluctuations rather than Brownian motion [16]. Let $e_{u}$ be the unit vector in the direction of motion, and the following equation describes the sperm cell’s motion:

(1) $$\dot{u}=v_{0}e_{u}\;,\;\;\dot{\theta }=\sqrt{2D_{\theta }}\;\xi \left(t\right).$$

At any time, the unit vector in the direction of motion is given by $e_{u}=\left[\cos\theta ,\sin\theta \right]$. The term ξ(t) represents Gaussian-distributed white noise. We use the Euler–Maruyama scheme for the actual numerical simulations: at every timestep, the angle θ is updated through the addition of a small random number. The actual ξ(t) is implemented by taking a set of Gaussian-distributed numbers that have zero average and unit variance. Subsequent values of $\xi (t_{i})$ are obtained by dividing these numbers by the square root of the timestep Δt.

The trajectories generated by Equation (1) display a persistence length $\ell_{p}=v_{0}/D_{\theta }$. The persistence length is a notion that is also well known in polymer theory [10,11,12]. We parametrize the trajectory with the contour length *ℓ*. Obviously, the direction θ changes as a function of *ℓ*. We consider the orientations $\theta (\ell_{0})$ and $\theta (\ell_{0}+\ell )$, i.e., the orientations at two points that are a distance *ℓ* away from each other along the contour. A good measure for the correlation between $\theta (\ell_{0})$ and $\theta (\ell_{0}+\ell )$ is the average 〈cosΔθ(ℓ)〉, where $\Delta \theta \left(\ell \right)=\theta \left(\ell_{0}+\ell \right)-\theta \left(\ell_{0}\right)$, and the average is taken over all positions $\ell_{0}$ along the entire trajectory such that the different segments of length *ℓ* do not overlap. For a very stiff, almost straight trajectory, 〈cosΔθ(ℓ)〉 is close to one for all *ℓ*. The persistence length $\ell_{p}$ is defined by $\langle \cos\Delta \theta \left(\ell \right)\rangle =\exp\left[-\ell /\ell_{p}\right]$ [10,11,12]. A smaller persistence length makes a trajectory appear more tightly wound (cf. Figure 2).

Related to the persistence length is the Kuhn length. Imagine a point *P* along the trajectory. Points that are $\ell_{p}$ to the left and right of *P* come with orientations that are not correlated to the orientation at *P*. With this realization, we can then imagine the trajectory as being built up from straight segments with a length of $\ell_{k}=2\ell_{p}$. At each junction, the angle between the linked segments is a random number from a flat distribution between 0 and 2π. The polymer or trajectory conceived as such is called a Freely Jointed Chain (FJC), and $\ell_{k}$ is known as the Kuhn length.

The FJC comes with concise formulae. For a chain of a total length *L* and with *N* Kuhn segments of length $\ell_{k}$, we have

(2) $$L=N\ell_{k}\;\;\mathrm{and}\;\;\langle S^{2}\rangle =N\ell_{k}^{2}$$

for the total contour length *L* and the average square end-to-end distance $\langle S^{2}\rangle$, respectively. These formulae are well known, widely applied, and explained in many authoritative textbooks, although mostly in the context of polymer theory [10,11,12].

For a searching particle that emerges at the center of the disk, it makes sense to search in such a way that any random point on the disk has an equal likelihood of being “found”. An annulus at a distance *r* from the center of width Δr covers an area 2πr(Δr) (cf. Figure 2). A search by a moving particle is thus more likely to be successful if the dwelling time at radius *r* is proportional to *r* itself. For an effective search, the trajectory should become tighter as the rim is approached, i.e., the Kuhn length $\ell_{k}$ should decrease during the search. A simple physiological course of action to achieve such a decrease in $\ell_{k}$ is to emerge from the center with an initial Kuhn length $\ell_{k,1}$ and a well-tuned transition rate to a smaller value $\ell_{k,2}$.

Consider the disk with radius r=1, as depicted in Figure 2. Figure 2a shows a search with $v_{0}=1$ and $D_{\theta }=2.0$, which implies a persistence length of $\ell_{p}=0.5$. Figure 2b depicts the realization of a search with an initial $D_{\theta ,1}=0.67$ and a transition rate k=2.0 to a final $D_{\theta ,2}=6.0$. Note that the $D_{\theta }=2.0$ in Figure 2a is the geometric average of $D_{\theta ,1}$ and $D_{\theta ,2}$.

The unit disk has three times as much area outside $r=\frac{1}{2}$ as it has inside $r=\frac{1}{2}$. It thus makes sense to explore the disk’s outer part ($r>\frac{1}{2}$) with three times the contour length of the inner part. After some algebra, it follows from Equation (2) that with a Kuhn length that is one-ninth of the original one, approximately the same end-to-end distance is covered with three times the contour length. Therefore, we take $D_{\theta ,2}=9D_{\theta ,1}$. Finally, it needs to be noted that, for all generated trajectories, we take Δt=0.001 for the timestep.

**Figure 3:**
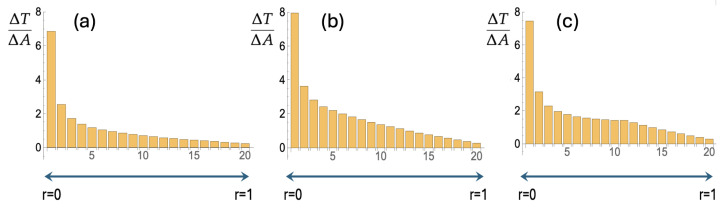
(**a**) Trajectories as in Figure 2a were generated, and for each region (one small circle and nineteen annuli), the time spent per unit area, ΔT/ΔA, was recorded. This figure depicts the averages over 5000 trajectories. (**b**) As for Figure 2b, we implemented a varying $D_{\theta }$: the searcher leaves the origin with a constant transition rate to the larger value of $D_{\theta }$. This leads to an improved search in that ΔT/ΔA becomes larger and more constant. (**c**) This figure shows how further improvement is achieved upon giving the searcher the ability to “read” its position and let the irreversible transition to the larger $D_{\theta }$ occur when r=1/2 is first traversed. Details are in the text.

In Figure 2, the unit disk is divided into nineteen annuli, each with a width of 1/20. In the center, there is one small circle with radius 1/20. A search is most effective if each location on the disk has an equal chance of being visited by the searcher. In terms of the annuli, this means that in each annulus, the searching particle spends the same amount of time per unit of area, i.e., ΔT/ΔA (cf. Figure 3) should be constant. Each of the three bar charts in Figure 3 is the result of 5000 trajectories generated with Equation (1). For each trajectory, the amount of time in each annulus was recorded and then divided by the area of that annulus. Figure 3 represents the average over 5000 trajectories for three cases. For Figure 3a, the radial diffusion coefficient was held constant at $D_{\theta }=2$. For Figure 3b, each of the 5000 searchers starts at the origin with $D_{\theta ,1}=0.67$ and a transition rate from k=2.0 to $D_{\theta ,2}=6.0$. Figure 3c depicts the result for searchers that can detect their own positions and irreversibly switch from $D_{\theta ,1}=0.67$ to $D_{\theta ,2}=6.0$ when r=0.5 is first traversed.

Going from Figure 3a to Figure 3b, we see an improved search. The average and standard deviations of ΔT/ΔA over the twenty regions go from 1.1 and 1.5 to 1.7 and 1.7, which implies that the relative standard deviation (the standard deviation divided by the average) goes from 1.3 to 0.98. Furthermore, the escape time $T_{\mathrm{esc}}$, i.e., the average time it takes to go from the center of the disk to r=1, increases from $T_{\mathrm{esc}}=1.8$ to $T_{\mathrm{esc}}=3.1$ in the setup with the switch of $D_{\theta }$.

There is another small improvement as we go from Figure 3b to Figure 3c, i.e., to a setup where the searcher “reads” its position and makes the switch contingent on the position. For the search in Figure 3c, the average and standard deviations are 1.6 and 1.5, resulting in a relative standard deviation of 0.94. The value of $T_{\mathrm{esc}}$ is the same as for the non-position-guided switch, i.e., $T_{\mathrm{esc}}=3.1$. Particularly striking in Figure 3c is how ΔT/ΔA stays constant in the region between about r=0.2 and r=0.7.

All in all, both switch-included searches last about 70% longer than the original search in Figure 2a and Figure 3a. Both of these enhanced searches are also more uniform in that among the annuli in the unit circle, the search times per area (ΔT/ΔA) have a relative standard deviation that is reduced by about 25% compared to the original switchless search. The improvements going from Figure 3a to Figure 3b and then to Figure 3c may appear small. However, in the 1930s, the “New Evolutionary Synthesis” combined genetics and population dynamics to put evolution on a more quantitative and scientifically rigorous foundation. It was found that even small selective advantages drive evolution. The authoritative textbook by D. Futuyma [17] summarizes it as follows: “… a character state with even a minuscule advantage will be fixed by natural selection. Hence, even very slight differences among species, in seemingly trivial characters such as the distribution of hairs on a fly or veins on a leaf, could conceivably have evolved as adaptations”.

## 3. The Evolution of the Probability Distribution on the Disk

The previous section involved an ordinary differential equation with a stochastic input at every timestep. The ensuing stochastic differential equation describes a trajectory of the diffusing particle. It is possible to formulate an equivalent partial differential equation that describes the time evolution of the particle’s probability distribution P(x,y,t). The underlying theory is covered in many standard textbooks [18,19,20].

The equation that rules the 2D diffusion on the disk is

(3) $$\partial_{t}P=D\nabla^{2}P,\;\;\mathrm{where}\;\;P=P\left(x,y,t\right).$$

Here, *D* and $\nabla^{2}$ stand for the diffusion coefficient and the Laplace operator, respectively. The diffusion coefficient *D* in this equation has a simple relation to the $D_{\theta }$ in Equation (1). For a particle diffusing in 2D, the mean square displacement and the elapsed time are related through $\langle \Delta r^{2}\rangle =4D\;\Delta \tau$. The Δr can be identified with *S* in Equation (2). The contour length *L* can be identified with $v_{0}\;\Delta \tau$. This leads first to $D=\frac{1}{4}\ell_{k}v_{0}$, and after invoking $\ell_{p}=\frac{1}{2}\ell_{k}$ and $\ell_{p}=v_{0}/D_{\theta }$, we find

(4) $$D=\frac{1}{2}\frac{v_{0}^{2}}{D_{\theta }}.$$

In polar coordinates and with circular symmetry, Equation (3) takes the form

(5) $$\partial_{t}P=D\left(\partial_{r}^{2}+\frac{1}{r}\partial_{r}\right)P,\;\;\mathrm{where}\;\;P=P\left(r,t\right).$$

Before further analysis, it is worth reflecting on Equation (5) in light of what was established in the previous section. The first term on the right-hand side of Equation (5) is a diffusion term that does not discriminate between the directions *r* and −r. The second term on the right-hand side is a drift term that contains a characteristic speed D/r. This may be puzzling as the diffusion should have no apparent direction. In his classic text, Feller [21] explained the apparent anisotropy as follows (Volume 2, Chapter 10, Section 6): “The existence of a drift away from the origin can be understood if one considers a plane Brownian motion starting at the point *r* of the *x*-axis. For reasons of symmetry its *abscissa* at epoch h>0 is equally likely to be >r or <r. In the first case certainly R(h)>r, but this relation can occur also in the second case. Thus the relation R(h)>r has probability $>\frac{1}{2}$, and on the average *R* is bound to increase”.

With an initial condition P(r,0)=δ(r)/(2πr) and on a disk with infinite radius, Equation (5) is readily solved by the spreading Gaussian:

(6) $$P\left(r,t\right)=\frac{1}{4\pi Dt}\exp\left[-\frac{r^{2}}{4Dt}\right].$$

This solution comes with a root-mean-square distance to the origin, $\tilde{r}$, which increases following ${\tilde{r}}^{2}=4Dt$. Taking the derivative with respect to time on both sides, we find that the speed of the root-mean-square distance $\dot{\tilde{r}}=2D/\tilde{r}$.

For a particle that drifts away from the origin with a speed v=2D/r, the time spent in an annulus of area ΔA=2πrΔr is found to be ΔT=(r/2D)Δr. So, the search time per area, ΔT/ΔA=1/(4πD), appears to be independent of *r*. This uniform coverage of the search area occurs with any drift speed that is proportional to 1/r.

However, it is not hard to see that such *r*-independence of ΔT/ΔA only works for a disk with a radius R→∞. If a diffusing particle is absorbed upon hitting the boundary at a finite r=R, any trajectory leading to a position r<R after passing through an r>R region is eliminated. This leads to a decreasing ΔT/ΔA as *r* increases. Such a decrease is indeed what we see in Figure 3. Below, we add mathematical rigor to these insights and observations.

An absorbing boundary at r=R means that P(R,t)=0. Solutions for setups with an absorbing boundary condition can commonly be found by placing an “image initial condition”, $\bar{P}\left(r,t=0\right)$, outside the disk. The image is negative, and the positioning of the image initial condition has to be such that the ensuing flow toward r=R (governed also by Equation (5)) leads to $\bar{P}\left(R,t\right)+P\left(R,t\right)=0$. The full solution $\left(P+\bar{P}\right)\left(r,t\right)$ then satisfies Equation (5) in the area r<R.

For 1D diffusion along an axis, the image method is easily implemented and readily leads to a solution. But complications arise in the 2D case. As previously mentioned in the context of Equation (5), the directions toward the disk’s center and away from the disk’s center are *not* equivalent. For an image that is initially positioned at r=2R, i.e., $\bar{P}\left(r,t=0\right)=-\delta \left(r-2R\right)/\left(4\pi R\right)$, a solution according to Equation (5) can be derived after some nontrivial mathematical operations [22]: $\bar{P}\left(r,t\right)=-1/\left(4\pi Dt\right)I_{0}\left(rR/(Dt)\right)\exp\left[-\left(r^{2}+4R^{2}\right)/\left(4Dt\right)\right]$, where $I_{0}$ represents the modified Bessel function of the first kind. The problem with this solution is that we do *not* have $\bar{P}\left(R,t\right)+P\left(R,t\right)=0$ at all times.

**Figure 4:**
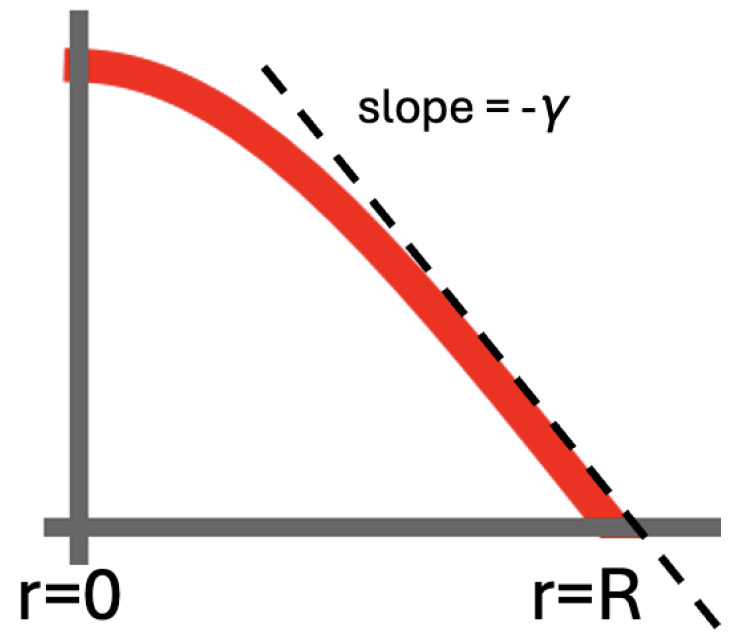
To the best of our knowledge, Equation (5) has no analytic solution on a disk with an initial condition P(r,t=0)=δ(r)/(2πr) and an absorbing boundary at a finite r=R. The absorbing boundary implies P(R,t)=0 at all times for the evolving P(r,t). We have $\partial_{r}P\left(R,t\right)=-\gamma$ for the slope at r=R. We take this as a first approximation also near the rim. This implies J(R,t)=Dγ for the amount of probability that crosses the circumference of the disk per unit of time and per unit of distance. With P(r,t)=γ(R−r) near r=R, we find v(r,t)=J(r,t)/P(r,t)=D/(R−r) for the drift speed toward the rim. Note that this result is independent of γ. The drift speed v(r)=D/(R−r) will thus remain at the same value during the entire relaxation.

Below, we derive some relevant features of the diffusion on the 2D disk without an actual closed-form solution. Through Fick’s Law, the outflow from the disk can be identified with $J\left(R,t\right)=-D\nabla P\left(R,t\right)=-D\partial_{r}P\left(R,t\right)$. If we set $\partial_{r}P\left(R,t\right)=-\gamma$ for the slope of the P(r,t) curve at r=R (cf. Figure 4), we have J(r,t)=Dγ at r=R. J(r,t)=Dγ is also a good approximation on r<R near the outer edge of the disk.

In our 2D case, the flux $J(r_{0},t)$ is the amount of probability that crosses at $r=r_{0}$ per unit of time and per unit of distance along the circumference of the circle with radius $r_{0}$. Next, it is the ratio $J\left(r_{0},t\right)/P\left(r_{0},t\right)$ that represents the average speed necessary to bring about this flux. With P(r,t)=γ(R−r), we obtain v(r)=D/(R−r) for the drift speed of the diffusing particle. We observe that, unlike the R→∞ case discussed earlier, the drift speed now increases as the absorbing boundary of the disk becomes closer. This can be readily intuited upon the realization that with r=R nearer, ever more possible trajectories hit r≥R and disappear from the domain.

As before, we divide our disk into annuli with a width Δr. With the drift speed derived in the last paragraph, we find that the dwelling time per annulus ΔT/Δr=(R−r)/D. From there, we derive a search time per area that depends on *r*:

(7) $$\frac{\Delta T}{\Delta A}\left(r\right)=\frac{R-r}{2\pi Dr}.$$

**Figure 5:**
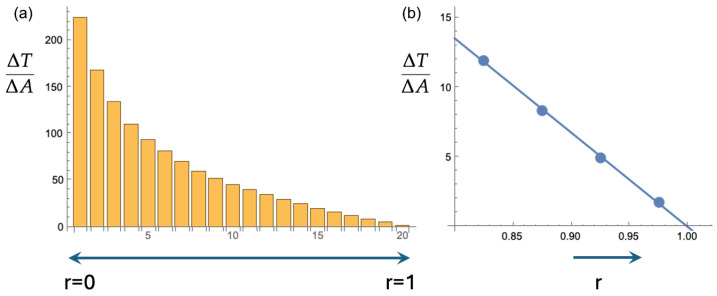
Equations (3) and (5) are only valid if $\ell_{p}\to 0$, i.e., if $D_{\theta }$ is very large. We simulate as in Figure 2 and Figure 3, but with $D_{\theta }=200$, leading to $\ell_{p}=0.005$. The figure on the left is the ensuing bar chart (cf. Figure 3). The figure on the right is a close-up view of the last four bars of the bar chart, together with the best linear fit. The slope of the fit is in good agreement with the prediction of Equation (7).

It is obvious that ΔT/ΔA vanishes as r→R. Figure 5 shows the results of a simulation. We let 1000 particles diffuse from the center of the disk to the absorbing boundary at r=1. The motion of the particles is again ruled by Equation (1), with $v_{0}=1$ and $D_{\theta }=200$. Through Equation (4), this leads to $D=2.5\times 10^{-3}$. The timestep used for the Euler scheme is again Δt=0.001. As before, the unit disk is segmented into a small r=0.05 disk and nineteen annuli with a width of 0.05. Like before, the twenty bars in the histogram (Figure 5a) give the average time per unit area spent in each of the twenty segments. Figure 5b gives ΔT/ΔA for the four outermost annuli, together with the best-fitting straight line. Equation (7) gives 63.7 for this slope. The slope of the fitted line is 67.7. The small discrepancy is not only due to the neglected curvature but also to the finite persistence length that is implicit when simulating Equation (1). In our case, $\ell_{p}=5\times 10^{-3}$. The partial differential equations that are central in this section, i.e., Equations (3) and (5), actually correspond to a fractal trajectory with zero persistence length.

A diffusion coefficient that decreases as the particle diffuses away from the center leads to a *D* that effectively decreases with *r*. As pointed out in the previous section, and as should now also be clear from Equation (7), this flattens out ΔT/ΔA as a function of *r* and leads to a more efficient search of the disk’s area.

Finally, it is worth pointing out that Equation (7) and the drift speed v(r)=D/(R−r) are independent of the actual slope −γ. This is a nice feature because as the particle diffuses away from r=0, the slope actually changes over time. Immediately after t=0, the slope increases. Later in the relaxation, the slope starts decreasing again. It is remarkable that Equation (7) and the drift speed v(r) remain the same throughout this process.

## 4. Discussion

We have studied a simple, bare-bones model to keep the analysis intuitive and to have manageable mathematics and numerics. Active particles can interact with each other in intricate ways (see, e.g., Ref [23]), but we have studied independent, non-interacting searchers.

**Figure 6:**
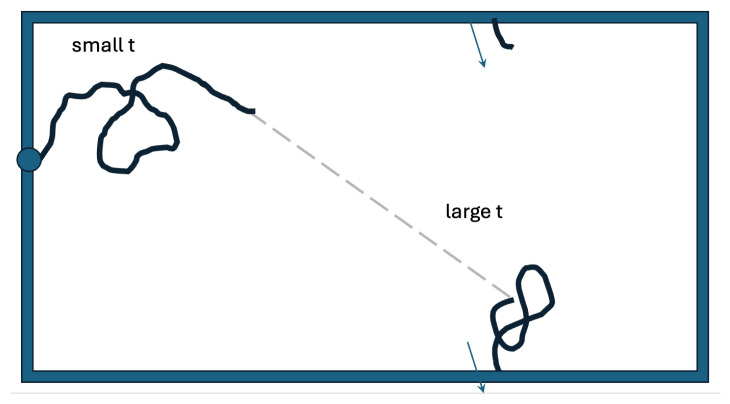
It is in the cylindrical oviduct that a sperm cell searches for the egg. This search can be modeled as a random walk that starts on the left and has an absorbing boundary on the right. The vertical coordinate is periodic. The geometry is a bit more complicated than this, as the oviduct’s surface has many twists and folds. Nonetheless, it makes sense for the searchers to first rapidly disperse and later switch to a smaller persistence length for a more detailed search.

Figure 6 presents a search space that more closely resembles an actual oviduct. In the oviduct, the searching sperm cells enter on one side. Here, we let them enter at an arbitrary point on the left edge. We account for being on the inside of a cylinder through the periodicity of the vertical coordinate. The infundibulum is the interface between the fallopian tube and the ovary. Sperm can “get lost” there, and we model this by implementing an absorbing boundary on the right edge. The two-state strategy we studied on the disk would also likely be effective in this case. A good dispersal of searchers could be achieved by taking off with a large persistence length in an arbitrary direction from an arbitrary point on the left edge. Each individual sperm cell would then perform a more detailed search after it switches to the smaller persistence length.

Reproductive biologists have been aware for a long time that sperm in the oviduct can switch to a less linear, more vigorous type of motion. They have termed this type of movement “hyperactivation” [13,24,25,26,27]. Many research efforts have been devoted to determining which types of chemicals can trigger the switch to hyperactivation. In terms of our Equation (1), hyperactivation corresponds to a larger $D_{\theta }$ and an ensuing smaller value of the persistence length $\ell_{p}$. It is commonly assumed that the purpose of hyperactive “wriggling” is detachment from the epithelium when the sperm cell is stuck there. In this work, we suggest that the switch to hyperactivation may simply be part of an optimized search strategy.

Over the past one and a half decades, an extensive literature about active Brownian particles has developed. Our Equation (1) commonly features in review articles on the subject as a fundamental model to which more effects can be added [28,29]. Some of the trajectories in Figure 1 exhibit chirality. Chirality has often been included in studies that model the motion of sperm cells [13] or other microswimmers [30]. In our minimal model, we did not take it into account. Chirality could readily be added to the model in Equation (1) through the addition of an angular velocity ω on the right-hand side of the second equation. However, such an addition would significantly complicate the subsequent analysis, and a derivation, such as that in Section 3, would be harder and less intuitive. It is obvious that chirality, or any added curvature for that matter, is like a smaller Kuhn length in that it leads to an area being more thoroughly searched. Nonetheless, the central result of our work, i.e., that a well-tuned switch to a much larger $D_{\theta }$ enhances the search, will only be affected if $\omega \gg D_{\theta }$ (see, e.g., Equation (3) in [13]).

Routine semen analysis in clinical practice relies on microscopic inspection of sperm characteristics and statistical comparison with fertile samples [31,32]. Contrary to common belief, fast motility alone is not necessarily an indicator of high fertilizing potential due to the complexity of the sperm search task and statistical variation among the large number of sperm present in an ejaculate [33]. Efficient search likely requires a combination of motility patterns, including high-persistence-length movement for rapid dispersal and low-persistence-length movement for thorough local exploration. Our modeling suggests that a well-tuned combination of both modes may enhance search efficiency on restricted domains such as those found in the complex environment of the female reproductive tract, where sperm must balance wide-area coverage with detailed exploration to locate the egg.

The two-state random walkers (Figure 2b and Figure 3b,c) that we studied represent improvements over the regular random walk (Figure 2a and Figure 3a). In closing, it is instructive to look at our subject matter and results from the wider perspective of computation and problem solving in biological systems. Searches are also part of macroscopic everyday reality. When a search-and-rescue team searches for a lost or missing person in a large wilderness area, a common strategy is to divide the search area into smaller areas. These smaller areas are labeled, and then different teams or individuals are assigned to different areas in the grid to conduct detailed searches. Obviously, sperm cells cannot achieve such a level of organization and coordination.

The random walk with identical searchers with a constant persistence length (cf. Figure 2a) is the most brute-force approach to the search problem. It is the Monte Carlo modus operandi and requires the mobilization of many searchers to achieve a non-negligible probability of fertilization. The random walk shown in Figure 2b and Figure 3b contains a transition to a smaller persistence length. We have shown that this feature leads to improved efficacy. Through the transition rate *k*, this search involves a characteristic time 1/k. So, the improvement is due to the usage of a very crude clock and the search being split up into a “before” and “after”. Figure 3c shows a bar chart corresponding to a random-walk searcher that does not have a clock but instead uses a very crude GPS. The searcher only “knows” whether it is in Region 1 or 2, and switches the persistence length the first time it “sets foot” in Region 2.

Biologically, a well-tuned *k* or an effective partitioning into two regions would be an outcome of natural selection. This improvement over the pure Monte Carlo algorithm can be viewed as enhanced agency and as something “learned”—a genetic legacy built up after much past “trial and error”. Neither of the two improvements involves any sperm-to-sperm communication.

The analogy with a team on a search-and-rescue mission underscores an important point. Popular culture often views ejaculate as a swimming contest. However, it is more appropriate to view ejaculate as tissue. A heart pumps, an ear detects sound, and ejaculate *searches*. Successful ejaculate is ejaculate that yields a high probability that one sperm cell finds and fertilizes an egg cell. It is obvious that there is selective pressure toward more successful ejaculate.

Sperm vary significantly in form and function within ejaculates, among individuals, and within individuals over time [34,35,36]. There is evidence that increased between-male competition for mates over time can drive apparent adaptations in sperm morphology and motility [37]. Simple features such as swimming velocity do not necessarily enable strong predictions about fertility competence when the individuals being compared are from the same species [38]. Additionally, the effect of sperm competition within an ejaculate remains unresolved [39]. Ultimately, a better understanding of the relationship between sperm form, function, and the physiological demands of the search task will enable these questions to be answered.

## Data Availability

Dataset available on request from the authors.
